# Impulsivity, impulsive aggression and borderline personality features among violent spouses

**DOI:** 10.1016/j.heliyon.2022.e10135

**Published:** 2022-08-13

**Authors:** Saba Kanwal, Syeda Farhana Kazmi

**Affiliations:** a Hazara University Manshera Dhodial, Department of Psychology, Pakistan; b Faculty of Psychology, Hazara University Manshera Dhodial, Pakistan

**Keywords:** Impulsivity, Impulsive aggression, Borderline personality features, Spousal violence

## Abstract

**Objective:**

Spousal violence in Pakistan is under researched but still not considered as a public health problem. The current study is intended to analyze the association between multiple measures of impulsivity, impulsive aggression, and borderline personality feature among violent spouses as well as to find out the moderating role of impulsive aggression between spousal violence and borderline personality features.

**Methods:**

A cross-sectional survey research design was used for conducting the research. The sample of 300 spouses, experiencing intimate partner relationships, was taken from different cities of the Hazara division through purposive sampling.

**Results:**

Present study demonstrated a significant positive correlation of spousal violence with impulsivity (r = .708∗∗, P < .001), impulsive aggression (r = .176∗∗, P < .001), and borderline personality features (r = .605∗∗, P < .001), while impulsivity was negatively correlated with impulsive aggression (r = -.018, P < .01). Impulsive aggression moderates the relationship between spousal violence and borderline personality features. The results showed that male and female spouses were found equally impulsive and impulsively aggressive. Likewise, impulsivity, impulsive aggression, and borderline personality features were found significant predictors of violence F = (3, 296) = 106. 67, P < .001.

**Conclusion:**

Current research offers some important insights and consequences for physicians and practitioners who interact with individuals who have experienced violence. These results have significant therapeutic implications for the treatment of violent couples.

## 1 Introduction

The problem of spousal violence in Pakistan is under-researched but still not considered a public health problem. Negative consequences of violence affect both victims as a result of injury or death and perpetrators as a result of imprisonment. In this study, the word “spousal violence” involves psychological and physical violence by a husband against his wife and vice versa. Psychological violence is acts of embarrassment, involuntary separation, coercion, etc., whereas physical violence is the use of physical force with the potential to cause injury, harm, or death and this involves kicking, punching, fire, and knife attacks.

This study will provide a step forward for investigating the link between multiple measures of impulsivity, impulsive aggression, and borderline personality feature among violent spouses. Furthermore, the relationship between these specific constructs opens up opportunities for multiple clinical measures, therapeutic interventions, and treatment strategies for violent spouses characterized by combinations of impulsiveness, impulsive aggression, and borderline personality feature.

Impulsivity is the key concept for thinking about the factors of violence and aggression (Edwards et al., 2003). Impulsivity is also characterized as a "predisposition towards sudden and unplanned reactions to internal stimuli or external stimuli with no regard to the negative consequences of such reactions on the impulsive individual or others" (Moeller et al., 2001), a term that is only acceptable for a personality trait, interpreted as a tendency to give a certain reaction to stimuli.

Impulsive aggression is the sudden and unexpected use of force or abuse by a person. This is the act of immediately responding aggressively to a trigger without having the time to understand the response or the repercussions. It is unplanned and presents as a disproportionate reaction to a perceived provocation (whether it be real or imagined) (Coccaro, 2015).

Borderline personality disorder is theoretically characterized by the existence of the following symptoms, according to the diagnostic criteria developed in DSM-5 (American Psychiatric Association [APA], 2013) modification of an individual's personality structure, intense distortions of self-image, persistent feelings of emptiness, behavioral changes, including suicidal attempts and self-harm, and excessive impulsivity. Borderline PD is characterized in DSM-5 as "stable and of long duration" (APA, 2013, p. 647) and "lifelong" (p. 665). The overall description of PD in DSM-5 Section III (Alternative DSM-5 Model for Personality Disorders) is that "impairments in personality functioning and the individual's personality characteristic expression are generally consistent through time.

Past study has found that people dealing with borderline personality disorder have experienced major impairments in their role as spouse/partner. South et al. (2008) found that borderline personality and antisocial personality disorder in spouses were more likely to demonstrate excessive verbal aggression than many other personality disorders. Individuals with borderline personality traits are especially vulnerable to negative emotion and aggression in reaction to social stressors. In a sample of 109 heterosexual couples (Maneta et al., 2013), people with a high degree of unstable behavior were more prone to conduct interpersonal abuse and no relationships were found in women. In contrast, Weinstein et al. (2012) proposed that in women borderline personality symptoms were more strongly related to intimate partner violence perpetration as compared to men. Oquendo and Mann (2000) in a study of the borderline personality literature describe impulsivity as a predisposition toward having a short dormancy to acting on urges and propose that the urges of most proven significance are those that can or do result in harm to self or others. The often-recognized key component of borderline personality disorder is impulsivity, although its precise definition is often correlated with violence.

A borderline personality disorder is associated with violence toward others and the self. Violence towards others was significantly characterized by impulsivity and severe rage, while violence towards oneself was significantly characterized by avoidance of abandonment, self-mutilation, feelings of loss, and severe rage (Harford et al., 2019).

Nedegaard et al. (2019) study conducted on the conceptualization of intimate partner violence and proposed that a variety of transitory variables may affect the person. One potential transitional variable is impulsivity. Findings indicate that low-impulsive individuals could be at higher risk of preferring violent conduct in situations of marital conflict. Ross and Babcock (2009) studied males who were violent toward their female partner concerning proactive and reactive violence and borderline personality disorder was related to reactive violence. It was hypothesized that the relationship between BPD and male offenders of spousal violence signifies that BPD is comparatively common in men with externalized aggression.

Researchers examined the link between BPD and IPV. Additionally, gender effects and relationships between BPD and other forms of IPV have been explored. The study sample consisted of 250 men and women recruited using a reputable crowdsourcing platform. Results indicate that BPD is significantly correlated with the occurrence of IPV (Munro and Sellbom, 2020). Previous research reported that Impulsivity is a strong predictor of interpersonal violence, aggression, and adjustment issues in male criminals. Rage, abusive personality traits, and impulsivity are stronger predictors of violence (Cunradi et al., 2009; McMahon et al., 2018; Rodriguez-Fornells et al., 2002).

The theory of reactive aggression focuses on emotional and neural mechanisms contributing to behavioural reactions. This theory states that when a person is faced with an unpleasant situation, the following events occur: (1) an aversive stimulus triggers a negative emotional reaction, (2) the negative emotional response causes an impulse to harm others or thoughts of harming others, and (3) the instinct to injure causes aggressive behaviour unless inhibiting factors are present (Berkowitz, 1993).

Several studies have proposed the theory of reactive conflict in family conflict. According to one report, a subset of men who harass their wives was known as "borderline/cyclical batterers." When these men experience or are confronted with real rejection or alienation by their partners, they have been known to respond with rage. When these men are in physical distress, they are overwhelmed by the need to hurt others, and they consider hurting their partner. If something appears to interrupt them, the urge and thought will be replaced by anger and aggressive behaviour toward their partners (Hyde-Nolan and Juliao, 2012).

### 1.1 Conceptual model of the study

**Figure 1:**
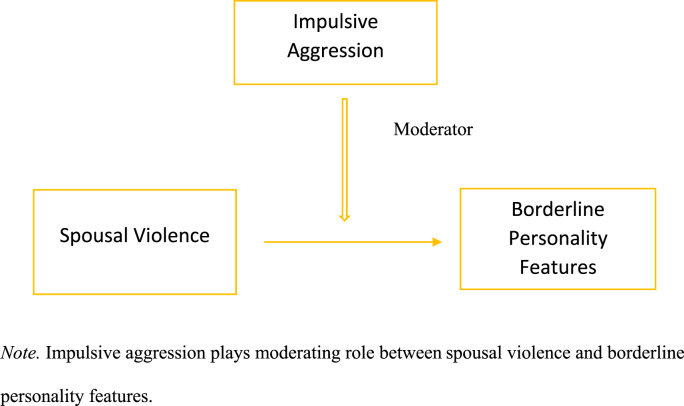
The conceptual model hypothesized in the current study focused on objectives and literature. *Note.* Impulsive aggression plays moderating role between spousal violence and borderline personality features.

Figure 1 shows that Impulsive aggression play moderating role between spousal violence and borderline personality features among violent spouses. Interaction between impulsive aggression and spousal violence strengthens the borderline personality features among violent spouses.

### 1.2 Objectives of the study

The objectives for this study are as follows:

1. To assess the level of impulsivity, impulsive aggression, and borderline personality features among violent spouses.
2. To formulate the moderating effect of Impulsive aggression on the relationship between spousal violence and borderline personality disorder.
3. To investigate the effect of gender on impulsive aggression, impulsivity, and borderline personality features of violent spouses.

### 1.3 Hypotheses of the study

The hypothesis of the current study is given below:

1. There will be a correlation among impulsivity, impulsive aggression, and borderline personality features of violent spouses.
2. Impulsive aggression will moderate the relationship between spousal violence and borderline personality features.
3. Male violent spouses will score high on impulsiveness and impulsive aggression as compared to female violent spouses.
4. Female violent spouses will have more inclination of borderline personality features than male violent spouses.

## 2 Methods

### 2.1 Research design

A cross-sectional survey research design is used for conducting the research.

### 2.2 Sample and procedure

In the present study, the purposive sampling technique is used to collect the desired data from the sample of 150 male participants and their female spouses who are living together for at least 12 months. Purposive sampling technique is used because researcher wants to collect first hand data from the sample. Female spouses and male spouses are characterized as violent if their partner reports on the Abusive Behavior Inventory and their cutoff scores are 10 or greater. A sample of violent spouses is obtained from the Hazara Division. We obtained informed consent from the participants, and the Research Ethics Committee of the Faculty of Psychology of the Hazara University approved this study. This study was performed according to the ethical principles of the Declaration of Khyber Pakhtunkhwa.

#### 2.2.1 Inclusion/exclusion criteria

Inclusion criteria involve only those spouses who have experienced spousal violence in the last six months. Participants who didn't experience spousal violence in the last six months are excluded from the study.

### 2.3 Instruments

#### 2.3.1 Demographic information form

The researcher has prepared a demographic information form to gather details on the age, gender, and education of the participants.

#### 2.3.2 Barrett's impulsiveness scale (BIS-11)

The impulsiveness scale of Barrett is used to describe the impulsiveness of the groups. The method is commonly used for the measurement of impulsivity (BIS-11). The Barratt Impulsiveness Scale (BIS-11; Patton et al., 1995) consists of 30 items. Items are graded on a scale of 4-points (1 = Rarely/Never, 2 = Occasionally, 3 = Often, 4 = Almost Always/Always). Cronbach's alpha was found to be among the 30 items, indicating good internal consistency as indicated by Cronbach's alphas ranging from .79 to .83 (Patton et al., 1995).

#### 2.3.3 Impulsive/premeditated aggression scales (IPAS)

The Impulsive/Premeditated Aggression Scale is planned to assess the average perception of one's aggressive behavior during the last six months (Stanford et al., 2003). It consists of 30 items in which 10 of the items (3,4,7,9,13,15,21,24,26,27) emphasize the characteristics of impulsive aggressive (IA) and 8 items (1,2,6,10,12,14,20,29) emphasize the characteristics of premeditated aggressive (PM). All objects are graded on a 5-point scale (5 is for Strongly Agree, 4 is for Agree, 3 is for Neutral, 2 is for Disagree, 1 is for Strongly Disagree). Higher scores have been indicated that individual has more impulsive aggression. The Cronbach's alpha for IPAS was .72–.82 (Stanford et al., 2009).

#### 2.3.4 Abusive behavior inventory

Spousal violence is measured using the Abusive Behaviour Inventory (ABI) created by Shepard and Campbell (1992) to quantify both psychological and physical assault. ABI contains 10 physical and 20 psychological violence items on a 5-point scale (0 is for never, 1 is for rarely, 2 is for occasionally, 3 is for frequently, 4 is for very frequently). The cutoff scores are 10 or greater. All the statements of the scale were calculated to find an overall ranging from 0 to 120 were increased in scores revealed the greater rate of violence. Cronbach's alpha for ABI was .92 (Shepard and Campbell, 1992).

#### 2.3.5 Personality assessment inventory-borderline scale (PAI-BOR)

The Personality Assessment Inventory-Borderline Features Scale (PAI-BOR) (Morey, 1991) comprises 24 items on a 4-point scale (0 = false to 3 = very true). The PAI-BOR includes four subscales and each subscale comprises 6 items that represent the main aspects of the BPD: affective instability, identity problems, negative relationships, and self-harm. The Cronbach's alpha for PAI-BOR was .82 (Morey, 1991).

### 2.4 Analysis and interpretation of data

In the current study the SPSS-25 is used to analyze the data. Correlation, multiple regression, t-test and moderation analysis are applied for the verification of the hypotheses.

## 3 Results

The current research found the relationship of impulsivity, impulsive aggression, and borderline personality features among violent spouses along with gender differences on these variables.

Table 1 shows that Barratt Impulsiveness Scale (BIS-11), Impulsive/Premeditated Aggression Scales (IPAS), Abusive Behaviour Inventory (ABI), and the Personality Assessment Inventory Borderline Features Scale (PAI-BOR) have .73, .81, .93, and .71 reliability. These alpha values are evidence of the internal consistency of these scales, that all the scales are reliable. Skewness values indicate that the data is normal.

**Table 1:** Psychometric properties of impulsiveness, impulsive aggression, abusive behaviour and borderline personality features (N = 300).

Scale	*n*	*M*	*SD*	*α*	Range		skew
					Potential	Actual	
BIS	30	61.83	9.48	.73	30–120	47–83	.573
IPAS	30	90.02	14.83	.81	30–150	68–125	.458
ABI	30	37.63	20.92	.93	0–120	10–95	1.06
PAIBOR	24	27.47	7.64	.71	0–72	17–44	.534

*Note,* Skew = Skewness, BIS = Barratt Impulsiveness Scale, IPAS = Impulsive/Premeditated Aggression Scales, ABI = Abusive Behaviour Inventory, PAI-BOR = Personality Assessment Inventory Borderline Features Scale.

Table 2 shows non-significant correlation between impulsivity and impulsive aggression (r = -.018, P < .01). The table also indicates a positive correlation between impulsivity and borderline personality features (r = .638∗∗, P < .001). Spousal violence is positivity correlate with borderline personality features (r = .605∗∗, P < .001), impulsive aggression (r = .176∗∗, P < .001) and impulsivity (r = .708∗∗, P < .001).

**Table 2:** Correlation among impulsiveness, impulsive aggression, abusive behaviour and borderline personality features (N = 300).

Variables	*1*	*2*	*3*	*4*	*M*	*SD*
1. BIS	-	.638∗∗	.018	.708∗∗	61.83	9.48
2. PAI		-	.136∗	.605∗∗	27.47	7.64
3. IPAS			-	.176∗∗	36.69	3.54
4. ABI				-	37.63	20.92

*Note,* BIS = Barratt Impulsiveness Scale, IPAS = Impulsive/Premeditated Aggression Scales, ABI = Abusive Behaviour Inventory, PAI-BOR = Personality Assessment Inventory Borderline Features Scale.

∗∗P < .001, ∗P < .05.

Table 3 show significant gender differences on, Abusive Behaviour Inventory (ABI) and the Personality Assessment Inventory-Borderline Features Scale (PAI-BOR) among violent spouses. A non-significant difference was found between Barratt Impulsiveness Scale (BIS-11) and Impulsive/Premeditated Aggression Scales (IPAS). The result of the study indicates that a male's spouse has an equal score on impulsivity (M = 63.24, SD = 9.64) and impulsive aggression (M = 38.16, SD = 3.41) than females spouses' impulsivity (M = 62.41, SD = 9.30) and impulsive aggression (M = 37.21, SD = 3.60). Results also indicate that female spouses experience a high level of violence (M = 52.30, SD = 20.30) and have more borderline personality features (M = 31.72, SD = 7.22) as compared to males spousal violence (M = 22.57, SD = 5.60) and male borderline personality features (M = 23.22, SD = 5.36).

**Table 3:** Mean differences along with gender on variables of impulsiveness, impulsive aggression, abusive behaviour and borderline personality features (N = 300).

Variable	Males		Females		*t* (298)	*p*	95% *CI*		Cohen's *d*
	(*n = 150*)		(*n = 150*)						
	*M*	*SD*	*M*	*SD*			*LL*	*UL*	
BIS-11	63.24	9.64	62.41	9.30	1.07	.28	-.98	3.32	0.08
IPAS	90.33	3.41	89.71	3.60	-.36	.71	-4.00	2.74	0.04
ABI	22.57	5.60	52.30	20.33	17.05	.00	26.30	33.16	1.97
PAI-BOR	23.22	5.36	31.72	7.22	11.57	.00	7.05	9.94	1.34

*Note.* CI = Confidential Interval, LL = Lower Limit, UL = Upper Limit, BIS = Barratt Impulsiveness Scale, IPAS = Impulsive/Premeditated Aggression Scales, ABI = Abusive Behaviour Inventory, PAI-BOR = Personality Assessment Inventory Borderline Features Scale.

### 3.1 Impulsive aggression moderating the relationship between spousal violence and borderline personality features

Moderation analysis was done using the Hierarchal Multiple Regression by Entering method, to check the moderating effect of Impulsive aggression on the relationship between spousal violence (independent variable) and borderline personality features (dependent variable). See Table 4 for details.

**Table 4:** Hierarchical multiple regression for moderation analysis among moderator impulsive aggression, spousal violence and borderline personality disorder (N = 300).

Variables	Model A		Model B		Model C	
	*Β*	ΔR^2^	*B*	ΔR^2^	*Β*	ΔR^2^
Spousal Violence	.61∗∗∗	.364	.592∗∗∗	.371	.547∗∗∗	.380
IA (Moderator)	-	-	.095∗	-	.124∗∗∗	-
The interaction term (ZE_SV∗ZIA)	-	-	4.39∗∗∗	-	.111∗	-
R^2^	.366		.375		.386	
F	171.98∗∗∗		89.03∗∗∗		62.31∗∗∗	
ΔF	171.98∗∗∗		4.252∗		5.485∗	

∗∗∗*p* < .001.

*Note.* = Spousal Violence; IA = Impulsive Aggression.

Table 4 shows the moderating effect of impulsive aggression on independent and dependent variable interaction. Three models have been created, i.e., Model A, B, and C. In the first level of multiple hierarchical Regression, spousal violence, an Independent Variable, has been entered against the dependent variable; borderline personality disorder. This model is termed Model A. In the next level, impulsive aggression (Independent Variables) has been entered against the dependent variable; spousal violence. In the final level, Exposure to spousal violence and impulsive aggression (Independent Variables), with interaction term of standard scores (ZE_CV∗ZCSE), entered against the dependent variable; borderline personality disorder. Analysis was generated.

Model A produces statistics; *R*^*2*^ = .364, *F* (1, 298) = 171.98, *p* < .001. Exposure to spousal violence accounts 36.4 % variance in borderline personality disorder, and having statistical significance (*p* < .001). Model B produces statistics; *R*^*2*^ = .371, *F* (2, 297) = 86.03, *p* < .001, resulting in .363 of variance, which creates 37.1 % increase in variance (Δ*R*^2^ = .095, *ΔF* (1, 297) = 89.03, *p* < .001). Model C produces statistics; *R*^*2*^ = .380, *F* (3, 296) = 67.31, *p* < .001, resulting in 38 % variance, which creates 4 % increase in *R*^*2*^ of Model B (Δ*R*^2^ = .124, *ΔF* (1, 296) = 62.31, *p* < .001).

Moderating Variable; impulsive aggression, increases *R*^*2*^ from .366 to .386, and the interaction term of standard scores of spousal Violence and impulsive aggression (ZE_CV∗ZCSE) increases *R*^*2*^ from .375 to .386. Variance increases from 36.4 % to 37.3 %, and finally 40 %, in three models Multiple Hierarchical Regression, while adding moderating variable and interaction terms. This shows the moderating role of impulsive aggression in exposure to spousal violence and borderline personality features interaction, thus confirming the hypothesis.

**Figure 2:**
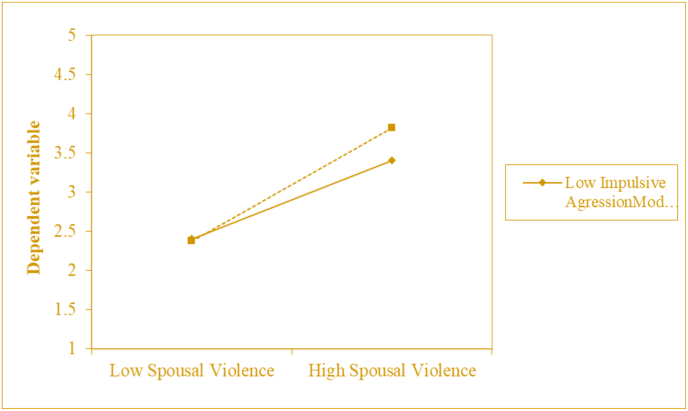
Moderating effect of impulsive aggression in relationship between spousal violence and borderline personality features.

As shown in Figure 2 significant positive correlation between the independent variable (spousal violence) and the dependent variable (borderline personality features). Impulsive aggression act as a moderator and has a significant association with borderline personality features. It indicates that violent spouses who scored high on impulsive aggression will have a strong relationship with borderline personality features.

Table 5 shows the impact of impulsivity, impulsive aggression, and borderline personality features on spousal violence. The R^2^ value of .51 revealed that the predictor explained 50% variance in the outcome variable with F = (3, 296) = 106. 67, P < .001. The finding revealed that impulsivity positively predicts spousal violence (β = .49, p < .001). Findings also show that borderline personality features a significant effect on spousal violence (β = .91, p < .001). Table also indicate significant effect of impulsive aggression on spousal violence (β = .06, p < .01).

**Table 5:** Multiple regression analysis for prediction of abusive behaviour (ABI).

Variables	ABI		
	Model 1		
	*β*	95%*CI*	
Constant (ABI)	39.2∗∗∗	[22.13, 56.28]	
BIS	1.1∗∗∗	[-1.37, -.89]	
IPAS	.02∗∗	[-.137, .098]	
PAI	2.5∗∗∗	[2.26, 2.84]	
*R*^*2*^	.51∗∗∗		
*F*	101.8∗∗∗		

*Note.* CI = Confidence Interval, BIS = Barratt Impulsiveness Scale, IPAS = Impulsive/Premeditated Aggression Scales, ABI = Abusive Behaviour Inventory, PAI-BOR = Personality Assessment Inventory-Borderline Features Scale.

## 4 Discussion

The Result of the present study showed a significant positive correlation between impulsivity and spousal violence (see Table 2). It is consistent with the results of previous research studies. Previous research studies have also provided evidence that Impulsivity is a strong predictor of interpersonal violence, aggression, and adjustment issues in male criminals. Rage, abusive personality traits, and impulsivity are stronger predictors of violence (Cunradi et al., 2009; McMahon et al., 2018; Rodriguez-Fornells et al., 2002).

Findings also indicate a strong positive correlation of impulsive aggression with spousal violence (see Table 2). The same results were determined by prior research. Lake and Stanford (2011) studied violent behavior and observed that impulsive aggression is considered an uncontrolled violent behavior, highly emotional, resulting from limited provocation. Impulsive batterers respond aggressively under high arousal conditions. Physical violence can often accompany a perceived provocation from his spouse or the suggestion that his spouse plan to quit and this inclination may be enhanced by personality patterns that promote dependency, vulnerability, rage, or emotional lability. Several reports have identified the use of impulsive aggression by a subset of perpetrators in this manner (Babcock et al., 2000; Merk et al., 2005; Cascardi et al., 2018).

According to current research results, there is a significant correlation between spousal violence and borderline personality features (see Table 2). Previous research studies have also determined that BPD is closely related to spousal conflicts, intimate violence, and a history of episodic interpersonal instability (Bouchard et al., 2009). South et al. (2008) studied married couples and found that borderline personality and antisocial personality disorder partners were more likely to engage in excessive abusive behavior than spouses with other behavioral conditions.

The results of the present study show non-significant relation between impulsivity and impulsive aggression. Previous research studies have also revealed that Impulsive–aggression had strong associations with measures of aggression and non-significant correlations with impulsivity. From a psychometric point of view, it, therefore, seems that the theoretical position most specifically operationalized by the main recommended constructive measure in BPD describes impulsive aggression as a subset of violent behavior which can be followed by impulsive characteristics for each person which for any individual may or may not also be accompanied by impulsive traits (Critchfield et al., 2004).

The current research study has determined that there is a positive correlation between impulsivity and borderline personality features. Previous research studies have also revealed that Impulsive behavior is seen as a central characteristic of borderline personality disorder. Impulsivity is considered a psychological and diagnostic characteristic of borderline personality disorder. Among all the symptoms of BPD, impulsivity is one of the symptoms that better describes borderline personality disorder. BPD has associated with impulsivity and severe rage (Harford et al., 2019; Henry et al., 2001; Sanislow et al., 2002; Sebastian et al., 2013).

Present study results also indicate significant gender differences in borderline personality features and spousal violence and non-significant gender differences in impulsivity and impulsive aggression (see Table 3). Current findings revealed that females have more borderline personality features than males. These results are consistent with previous research that has demonstrated that BPD is a global public health problem of considerable scope and concern. BPD is particularly harmful to women, who are three times more likely than men to be diagnosed with the disorder Skodol et al. (2005); Goldenson et al. (2007). Current research findings of the study do not support the hypotheses and the study concluded that male and female spouses were found equally impulsive. While previous research, about less violent and non-violent husbands, reported that extremely violent men were more impulsive, as stated either by themselves or their wives on a single questionnaire, and as measured by the results of a single test of behavioral impulsivity (Hancock et al., 2010). Current research findings of this study do not support the hypotheses and the study concluded that male and female spouses were scored equally on impulsive aggression. While prior research studies revealed that violent males show more impulsive aggression than females (Chapple and Johnson, 2007). The cultural and regional differences seemed to be responsible for the contradiction between current research findings and hypothesis.

Results revealed that impulsive aggression moderates the relationship between spousal violence (IV) and borderline personality features (DV) (see Table 4). Multiple Hierarchical Regression revealed 39% variation caused in borderline personality features due to the interaction of spousal Violence and impulsive aggression. According to prior research studies, impulsive aggression tests strongly reinforce the notion of elevated impulsive behavior of borderline personality disorder (Sebastian et al., 2013). It has also been determined that impulsive aggressions with measures of spousal violence strengthen borderline personality features (Critchfield et al., 2004).

The result of the study is consistent with the result of previous research and it is found that impulsivity, borderline personality features, and impulsive aggression positively predict spousal violence (see Table 5). According to the results of previous research, Impulsive aggression and impulsivity have been significantly associated with physical aggression (Edwards et al., 2003). The findings of the BPD profile were linked to both general and intimate partner abuse (Peters et al., 2017). A previous study has also found that borderline personality characteristics are favorably associated with physical and psychological violence (Armenti and Babcock, 2021).

### 4.1 Limitation and suggestion

The current study contained some limitations and suggestions that are listed below:

In the current research the sample was taken from limited regions of Pakistan; (Abbottabad and Mansehra) so for the future, it is suggested to take the sample from various other areas of Pakistan, for the full representation of the Pakistani spousal violent population. The present study has just explored the limited demographic differences (age, gender, level of education, and marriage duration) on impulsivity, impulsive aggression, borderline personality features, and spousal violence, and ignoring some other important demographics (e.g. socioeconomic status, family size, and cultural effects i.e. individualistic and collectivist culture) which have a strong impact on violence among spouses.so it is recommended, that in future research these demographics should be taken. In the current analysis, we take only one personality disorder, borderline personality disorder, and neglect other disorders that have a greater effect on spousal abuse, so it is recommended that other personality disorders e.g., antisocial personality disorder should be taken into account in future studies.

### 4.2 Implications

A significant contribution has been done by this study in a different area of psychology such as applied forensic and clinical work. Current research offers some important insights and consequences for physicians and practitioners who interact with individuals who have experienced violence. These results have significant therapeutic implications for the treatment of violent couples. They can help therapists identify complex psychological processes used by people in violent relationships. Previous researchers generally focus on the relation between impulsivity, impulsive aggression, and borderline personality features rather than identifying their role in spousal violence. While this research enables the researchers to find out the impact of treatment setting on assessment of the spousal violence.

## 5 Conclusion

The present study concluded that spousal violence was found significantly correlated with impulsivity, impulsive aggression, and borderline personality features of the spouses. It was found that impulsive aggression moderates the relationship between spousal violence and borderline personality features. Results indicate that a strong positive correlation exists between spousal violence and borderline personality features and this relationship has been moderated by impulsive aggression when spousal violence correlates with impulsive aggression in the form of interaction it caused to strengthen the borderline personality features among violent spouses. The overall finding suggests that impulsivity, borderline personality features, and impulsive aggression are a positive predictor of spousal violence and has a significant effect on spousal violence. Further studies into this field will take new directions. Overall, the findings can assist physicians, married partners, forensic investigators, and others in properly handling spousal conflict by reducing impulsivity, impulsive aggression, and borderline personality characteristics.

## Declarations

### Author contribution statement

Saba Kanwal: Conceived and designed the experiments; Performed the experiments; Analyzed and interpreted the data; Contributed reagents, materials, analysis tools or data; Wrote the paper.

Syeda Farhana Kazmi: Conceived and designed the experiments; Wrote the paper.

### Funding statement

This research did not receive any specific grant from funding agencies in the public, commercial, or not-for-profit sectors.

### Data availability statement

Data will be made available on request.

### Declaration of interest's statement

The authors declare no conflict of interest.

### Additional information

No additional information is available for this paper.
